# Effect of *Elodea nuttallii* Roots on Bacterial Communities and MMHg Proportion in a Hg Polluted Sediment

**DOI:** 10.1371/journal.pone.0045565

**Published:** 2012-09-17

**Authors:** Nicole Regier, Beat Frey, Brandon Converse, Eric Roden, Alexander Grosse-Honebrink, Andrea Garcia Bravo, Claudia Cosio

**Affiliations:** 1 F.-A. Forel Institute, Geneva University, Versoix, Switzerland; 2 Swiss Federal Research Institute WSL, Birmensdorf, Switzerland; 3 Department of Geoscience, University of Wisconsin-Madison, Madison, Wisconsin, United States of America; Laurentian University, Canada

## Abstract

The objective of this study was to assess the effect of a rooted macrophyte *Elodea nuttallii* on rhizosphere bacterial communities in Hg contaminated sediments. Specimens of *E. nuttallii* were exposed to sediments from the Hg contaminated Babeni reservoir (Olt River, Romania) in our microcosm. Plants were allowed to grow for two months until they occupied the entirety of the sediments. Total Hg and MMHg were analysed in sediments where an increased MMHg percentage of the total Hg in pore water of rhizosphere sediments was found. *E. nuttallii* roots also significantly changed the bacterial community structure in rhizosphere sediments compared to bulk sediments. *Deltaproteobacteria* dominated the rhizosphere bacterial community where members of *Geobacteraceae* within the *Desulfuromonadales* and *Desulfobacteraceae* were identified. Two bacterial operational taxonomic units (OTUs) which were phylogenetically related to sulfate-reducing bacteria (SRB) became abundant in the rhizosphere. We suggest that these phylotypes could be potentially methylating bacteria and might be responsible for the higher MMHg percentage of the total Hg in rhizosphere sediments. However, SRB were not significantly favoured in rhizosphere sediments as shown by qPCR. Our findings support the hypothesis that rooted macrophytes created a microenvironment favorable for Hg methylation. The presence of *E. nuttallii* in Hg contaminated sediments should therefore not be overlooked.

## Introduction

Mercury's nature: volatile, long-range transportable, bio-magnified and toxic, makes its use and release a global problem found everywhere on Earth, irrespective of the absence of local emission sources [1]. The toxicological concerns regarding Hg have given rise to extensive studies regarding its distribution and speciation in freshwater environments. Special attention has been paid to monomethylmercury (MMHg) because of its toxicity [2] and huge biomagnification in aquatic food webs [3]. MMHg can be formed in several abiotic and biotic processes, but the largest source is thought to be through transformations that are mediated by bacteria in aquatic sediments particularly sulfate-reducing bacteria (SRB) [3]–[7].

Shallow waters including lakes and rivers are known to provide a good environment for the formation of MMHg [8], [9] as well as for the development of macrophytes [10]. Bioaccumulation of Hg in macrophytes is possible under these conditions and has been regularly documented [11], [12], but on the other hand the influence of macrophytes on MMHg production is unclear. Hg resistant bacteria have been found associated to *Elodea* spp [13]. It was postulated that these bacteria protected the macrophytes from Hg by volatilizing it before entry into the cell [13]. Macrophytes have also been described to reduce the amount of dissolved Hg through the generation of organic ligands while also providing an environment suitable for demethylation [14]. On the opposite, a higher MMHg concentration in sediments colonized by macrophytes has been observed in salt marshes and wetlands [15], [16]. Previous studies have also shown that MMHg production is very high in roots of floating macrophytes in the Amazon and data indicated that SRB and other root-associated materials were the main responsibles for Hg methylation in this environment [17], [18]. In sum, the effect of macrophytes on Hg cycle is not yet well understood. It is not sure, for example, that data found in peculiar ecosystems such as salt marches or roots of floating macrophytes of the Amazon can be generalized to other ecosystems and/or plants. The rhizosphere is an ideal microhabitat for bacterial proliferation which in turn is important for plant growth and development. However, more studies are needed to understand the link between macrophyte presence and Hg methylation, in particular in freshwater environments of temperate climates.

The present work was conducted with sediments from a Hg contaminated site of the Olt River in Romania. Water, sediments and biota from the Babeni reservoir are heavily contaminated with Hg originating from a local chlor-alkali industrial plant [19]. Methylation of Hg occurs in this reservoir [20]. The highest MMHg concentrations in water were found in the deepest part of the reservoir near the dam [20]. However, the MMHg percentage of the total Hg (THg) significantly increased along the part of the reservoir colonized by macrophytes (upstream to middle part) [20]. *Elodea nuttalli*, an invasive submersed macrophyte was found to be well present in this part of the reservoir and in addition showed a significant accumulation of Hg (up to 2 mg/kg) and MMHg (28% of the THg) [21], [22]. We wondered if macrophyte, and in particular the presence of *E. nuttallii* could favor Hg methylation. Since methylation of Hg is thought to occur primarily in sediments, the present work was designed to assess the effect of *E. nuttallii* roots on bacterial communities and associated formation of MMHg in sediments. This work also allowed gaining basic information on the potential effect of macrophytes found in Hg contaminated sites on Hg cycle in freshwater temperate ecosystems.

**Figure 1:**
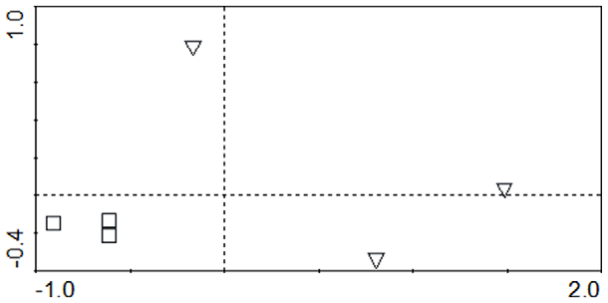
Principal component analysis (PCA) of rhizosphere (triangle) and bulk (square) communities of sediments. PC1 explained 73.9% and PC2 13.7% of the variation.

**Figure 2:**
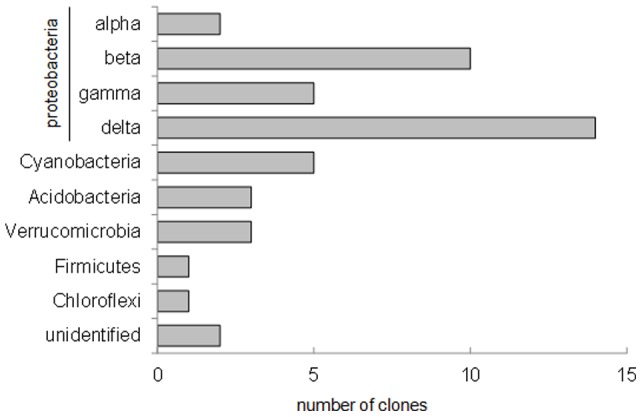
Abundance of bacterial taxonomic groups in the clone libraries of rhizosphere sediments (46 clones in total).

**Figure 3:**
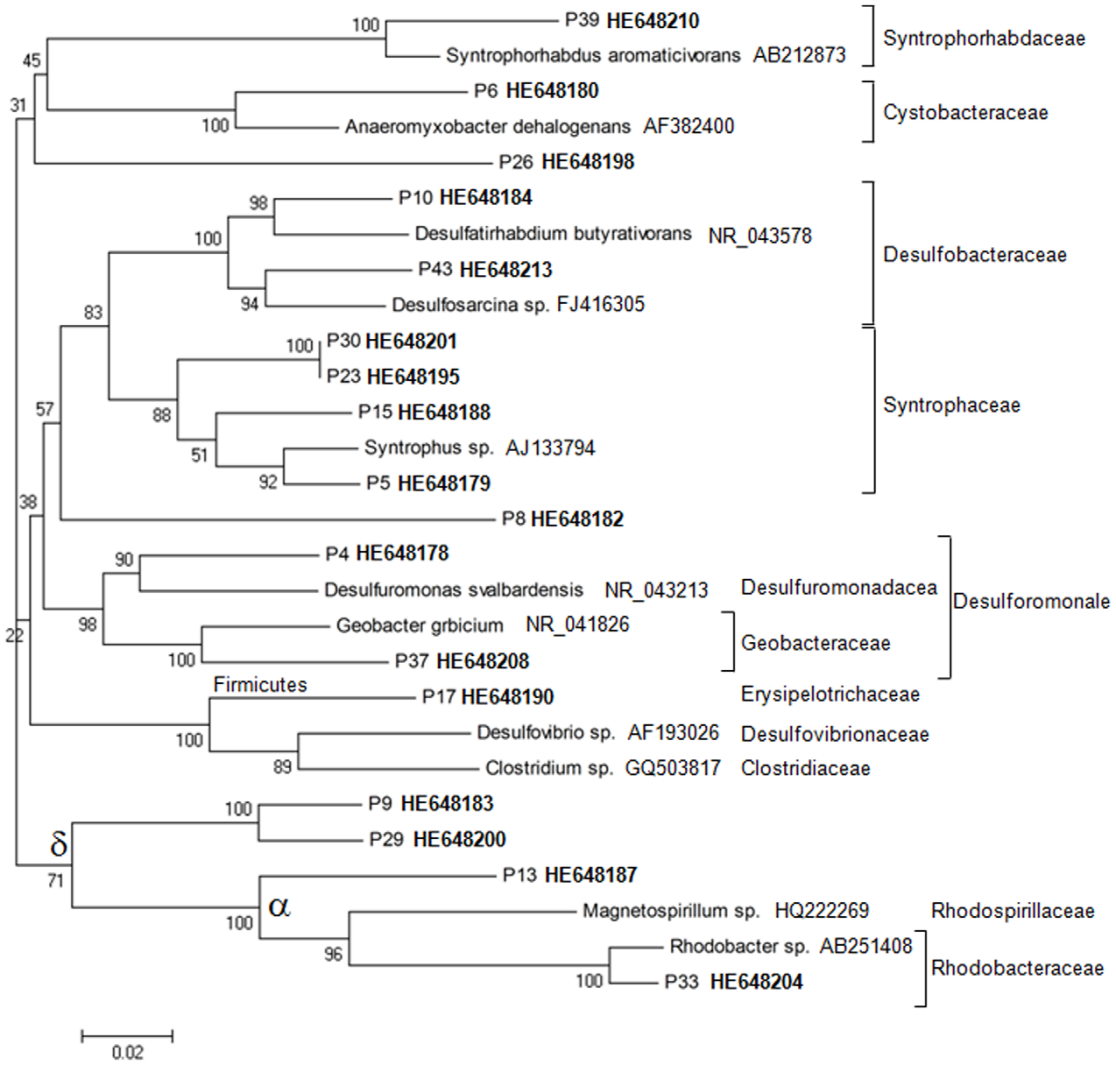
Unrooted neighbour-joining phylogenetic tree showing affiliation of clones (in bold) to closest related reference sequences of *Firmicutes, Alphaproteobacteria* and *Deltaproteobacteria* retrieved from rhizosphere sediments based on 16S rRNA gene sequences (1517 **bp). Bootstrap values are shown (500 replicates).**

**Figure 4:**
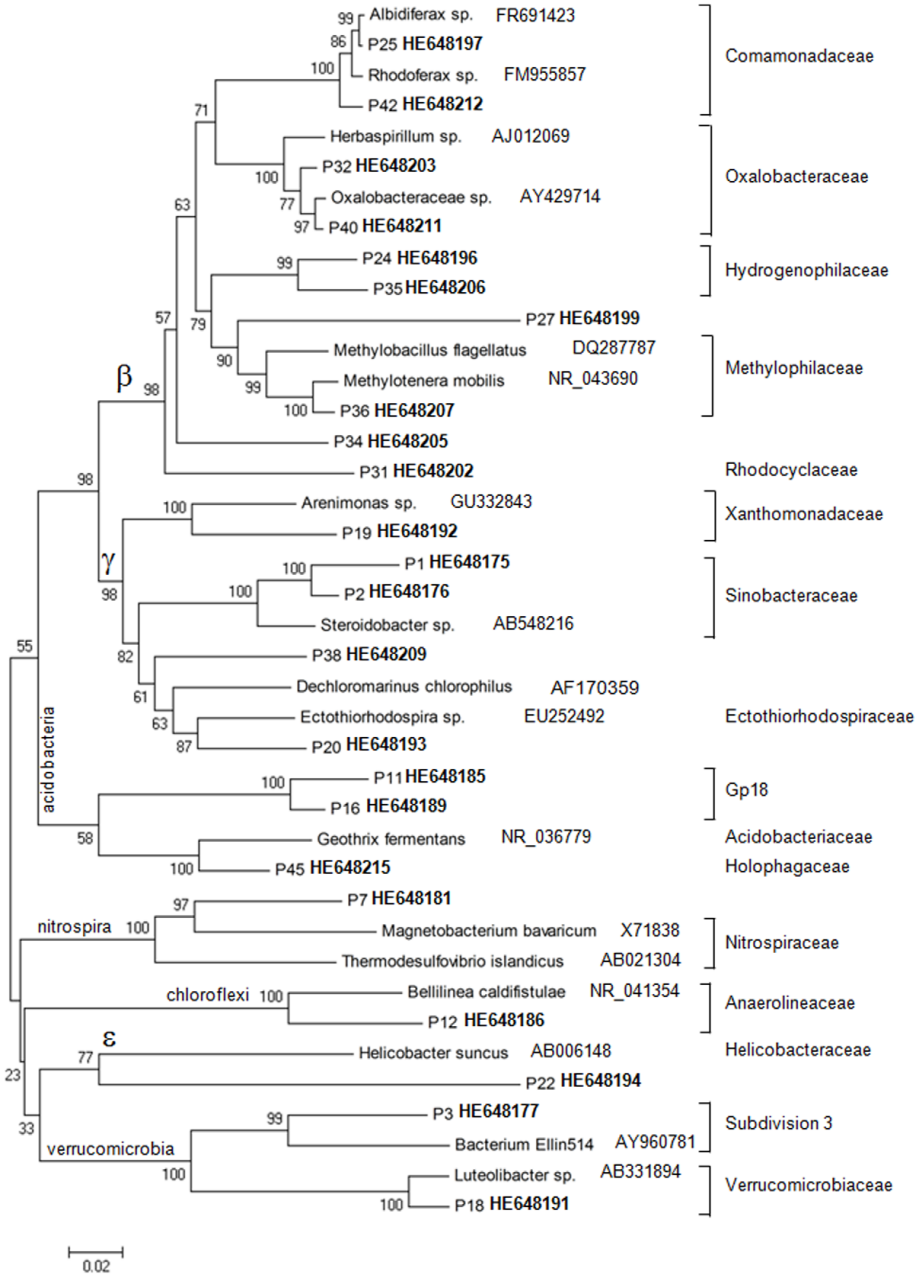
Unrooted neighbour-joining phylogenetic tree showing affiliation of clones (in bold) to closest related reference sequences of *Acidobacteria, Betaproteobacteria, Chloroflexi, Epsilonproteobacteria, Gammaproteobacteria, Nitrospira* and *Verrucomicobia*, retrieved from rhizosphere sediments based on 16S rRNA gene sequences (1517 **bp). Bootstrap values are shown (500 replicates).**

## Materials and Methods

### Sediments and plants sampling

Shoots of *Elodea nuttallii* and sediments were collected from the Babeni reservoir in August 2009. No specific permits were required for the described field studies: the location is not privately-owned or protected in any way and the field studies did not involve endangered or protected species. Sediments were collected with a surface grabber from the deepest part of the reservoir: a non-plant colonized area with high MMHg concentrations at the water/sediment interface [20]. Sediments were kept at 4°C until used. Shoots were acclimated to microcosm conditions and grown (20°C; 16∶8 hours light-dark cycle; 1000 lux) in sediments from Lake Geneva (_T_Hg 0.2±0.01 μg g^−1^, MMHg 0.002 μg g^−1^; pore water _T_Hg 0.25 ng L^−1^, MMHg was below detection limit 3SD_blank_ = 0.008 ng L^−1^) until used. Accumulation of Hg in plants growing on Lake Geneva sediments and effect on Hg in sediments was measured. A small increase of _T_Hg in plants was observed from 0.02 to 0.05 μg g^−1^, while MMHg was always below the detection limit. No effect was evidenced on Hg and MMHg concentration background in sediments and porewater of Lake Geneva sediments.

**Figure 5:**
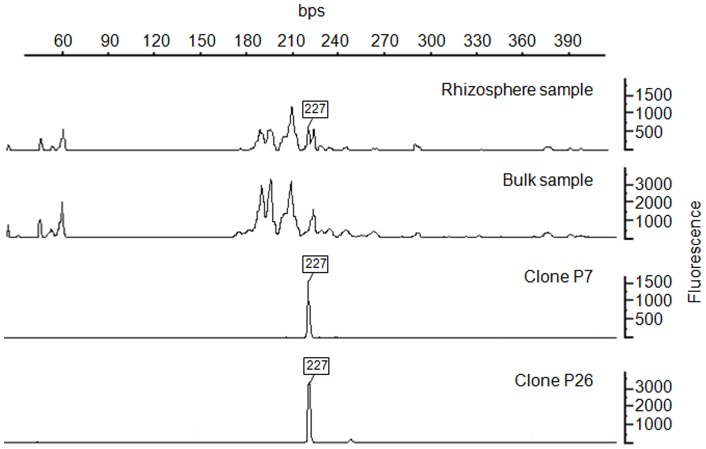
An experimental T-RF of clone P7 and P26 match one of the peaks increased in rhizosphere compared to bulk sediment.

**Table 1:** Quantification of bacterial 16S rRNA and *dsrA* gene copies in bulk and rhizosphere sediments by qPCR (gene copies/g dry sediment; mean ± std; n = 6).

Sediments	Universal	*dsrA*
Bulk	3.74±1.24*10^9^	2.02±0.80*10^6^
Rhizosphere	2.94±0.74*10^9^	1.39±0.70*10^6^

For the present work, three cm of Babeni reservoir's Hg contaminated sediments were put in 3 L pots. Pots were then filled with 1.2 μm filtered water from Lake Geneva, which was constantly renewed (total volume of water changed in 36 h) to minimize changes in water physico-chemistry over time that would affect plant growth. After one week of equilibration, three 5 cm-long shoots without roots of *E. nuttallii* were planted (or not: bulk sediments) in sediments and grown for 2 months until roots occupied the entirety of the available sediments. After 2 months, pots were transported into a glove box under N_2_ atmosphere, where plants with their roots were delicately removed and sediments were collected. Pore water was collected from the fresh sediments by centrifugation (5000 g for 45 minutes) and filtered at 0.45 μm under N_2_ atmosphere. Samples were immediately acidified to 0.5% HCl. Sediments for Hg analysis and DNA extraction were immediately freeze-dried and homogenized with an agate mortar. Concomitantly, plants were separated in roots and shoots and were washed for 5 min in milliQ water, followed by 5 min 1 mM EDTA, and 5 min in milliQ water. Thereafter, the plants were dried carefully with tissue before freeze-drying them for 24 h. All experiments were performed in triplicate.

**Table 2:** Concentrations of Hg and MMHg in pore water and sediments before (initial) and after two months of growth of *E. nuttallii* (bulk and rhizosphere) (mean ± std; n = 3; sign. a = 2α<0.05).

	Pore water		Sediments	
	Total Hg	MMHg	Total Hg	MMHg
Sediments	(ng/L)	(ng/L)	(μg/g)	(ng/g)
Initial	79±7	0.7±0.1	3.3±0.1	7,45±0,65
Bulk	81±8	0.7±0.1	3.4±0.03	7,57±0,75
Rhizosphere	42±4^a^	0.9±0.2	3.2±0.04	8.07±0.87

### Hg analysis

All materials and consumables were washed with acids (2 baths of 10% HNO_3_ and one bath of 10% HCl) before use to allow ultraclean sampling.

_T_Hg concentrations in sediments and plants prepared as described above were determined using an Advanced Hg Analyser AMA 254 (Altec s.r.l., Czech Rep). The accuracy of the measurements was checked by analyzing the certified reference material (CRM) Mess-3.

MMHg in sediments and plant samples was extracted by HNO_3_ leaching/CH_2_Cl_2_ extraction and analyzed by ethylation onto Tenex® traps followed by GC separation according to [23]. The accuracy of MMHg measurements was tested by analyzing the CRM ERM-CC580.

_T_Hg in pore water was measured by cold vapour atomic fluorescence spectrometry (CV-AFS) [24]. The accuracy of Hg measurements was tested by analyzing the CRM ORMS-3.

MMHg in pore water was measured by the hydruration method with cryogenic trapping, gas chromatography and atomic fluorescence spectrometry (CT-GC-AFS) [25].

For all Hg analysis methods, the analytical quality was assured by analyzing blanks and analytical triplicates within ±10%. For all methods the recovery of CRMs was at least 89%.

### T-RFLP analysis and clone library construction

DNA was extracted in triplicates from freeze-dried homogenized sediments with the Power soil kit (Mo-Bio) according to instructions of the providers. DNA was quantified spectrophotometrically and the concentration adjusted to 5 ng μl^−1^ with water containing bovine serum albumin molecular biology grade (Fluka) at a final concentration of 3 μg ul^−1^ and heated for 5 min at 90°C to bind PCR inhibiting substances such as humic acids [26]. Amplification of bacterial 16S rRNA genes was performed with primers 8F-1489R using Takara Ex Taq HS (94°C for 3 min and then 35 cycles 94°C 30 sec, annealing temp 52°C 30 sec, 72°C 1 min and final elongation 72°C 10 min) on 10 to 50 ng of purified DNA and according to provider instructions. Amplification was verified by electrophoresis of aliquots of PCR products on a 1% agarose gel. The highest concentration giving a specific PCR product was then the DNA concentration used for T-RFLP analysis and clone library construction.

For T-RFLP analysis we used the same primers on each DNA extract (n = 3) except that the forward primer 8F was fluorescently labeled. 1 μg of PCR products were digested by *Hae*III for 16 h (Fermentas) at 37°C. Digests were purified with QIAquick PCR Purification Kit (Qiagen) and digestion was verified by electrophoresis of aliquots on 1% agarose gel. Digestion products were prepared for analysis by capillary electrophoresis as follows: 3 μL of the digestion products were added to 11.4 μL of HIDI formamide (Applied Biosystems) and 0.6 μL of ROX500 DNA fragment length standard (Applied Biosystems). The samples were then denatured for 2 min at 95°C and immediately chilled on ice. Electrophoresis was performed for 30 min at 60°C with an ABI Genetic Analyzer 310 (Applied Biosystems) with 36 cm capillaries filled with POP-4 polymer [27].

The lengths of fluorescently labelled T-RFs were determined with internal standards using GeneScan 3.1. and Genotyper softwares (Applied Biosystems) with peak detection set to 50 fluorescence units. The size of the terminal restriction fragments (T-RF) given in relative migration units (rmu) and peak heights were determined with the GeneScan analysis software version 3.1 (Applied Biosystems). Peak signals were converted into numeric data for fragment size and peak height by using the Genotyper 3.7 NT (Applied Biosystems). The peak heights were recorded and compiled in a data matrix for statistical analysis. T-RF peak heights were normalized by dividing the peak heights of the single T-RFs by the sum of the total peak heights of all T-RFs according to [28], [29]. Significant T-RFs were defined as peaks with a size of *x*±0.5 relative migration units (rmu) and a height of at least 50 fluorescence units in all the replicates of at least one of the treatments. T-RFLP was used to appreciate the changes in the community structure, considering that distinct phylotypes could produce T-RFs of the same size and that estimation of diversity would therefore not be accurate [29], [30]. Average (*n* = 3) relative abundances (i.e. peak height) of each T-RF in each treatment were then compared for significant differences with the controls by one-way ANOVA corrected with the Tukey *post hoc* test (SYSTAT 10). The data from the T-RFLP profiles were further treated using Fisher's *Z* transformation. Principal component analysis (PCA) based on covariance was performed on the transformed data using CANOCO 4.5 software (Microcomputer power, Ithaca, NY).

For clone library construction, PCR products from the same DNA extracts as used for T-RFLP (see above) were pooled (n = 3), cloned in TOPO vectors (Invitrogen) and sequenced. Assignation of individual OTU to clone sequence was performed with the ribosomal database project classifier tool (97% cut off; http://rdp.cme.msu.edu/classifier/classifier.jsp). NCBI Blast (http://www.ncbi.nih.gov) was used to identify the most closely related 16S rRNA gene sequences. The 16S rRNA gene sequences were then all aligned in the Clustal W implementation of Bioedit (Ibis Bioscience). MEGA program was subsequently used to produce unrooted neighbour-joining phylogenetic trees (Kimura-2 correction; bootstrap values for 500 replicates) [31]. All the sequences described in this study have been submitted to the EMBL database under the accession numbers HE648175 to HE648215.

### Quantitative PCR (qPCR) of bacterial and dsrA gene copies

DNA was extracted from the freeze-dried homogenized sediments with the Power soil kit (Mo-Bio) according to instructions of the providers (n = 6). DNA was quantified spectrophotometrically. qPCR was conducted using a Bio-Rad iCycler™ equipped with a Bio-Rad iQ5™ multi-color real-time detection system, in conjunction with the Bio-Rad iQ5™ System Software (version 2.0, Bio-Rad). Bio-Rad iQ^TM^ SYBR® Green Supermix was used to perform the reactions according to the manufacturer's protocol. The universal bacterial primer set p338f/p518r was used to target the 16S rRNA gene, and the dissimilatory sulfate reductase gene (*dsrA*) was targeted using the primer set RH1-dsr-F/RH3-dsr-R. The sequences of these primers and the appropriate cycling conditions have been previously described [32]–[34], but amplifications in the current study were conducted with some modifications. A 30 s extension step at 72°C was added when using the RH1-dsr-F/RH3-dsr-R primer set, resulting in a three-step amplification protocol appropriate for use with the iQ^TM^ SYBR® Green Supermix. The previously described denaturing, annealing and extension temperatures for the p338f/p518r primer set were not modified, but the denaturing time in each cycle was decreased to 10 s, and the annealing and extension times were decreased to 30 s. The final 10 min extension step was omitted. All qPCR reactions were cycled 40 times, and real-time data was collected during the annealing step of each cycle. To verify qPCR specificity, software-generated melt curves were analyzed, and the reactions were run on a 1% agarose gel after completion (data not shown). Appropriate standard curves, positive, negative, and no-template controls were run with each reaction in duplicate. One microliter of sample, control or standard DNA was used in each qPCR reaction, and the numbers of targets per sample were calculated. All reactions were set up in a sterile PCR hood to limit potential contamination. The threshold cycles (C_q_) of no-template controls were noted, and any unknown sample with a greater C_q_ was not included in the final analysis.

PCR amplicons were used to construct standard curves for both qPCR primer sets. Genomic DNA was extracted from *Escherichia coli* DH5α and *Desulfovibrio vulgaris* subsp. *vulgaris* (ATCC 29579) using an UltraClean® Microbial DNA Isolation Kit (Mo-Bio). The manufacturer’s protocol was followed with one modification: prior to bead beating, samples were heated with shaking at 65°C for 10 minutes at 14'000 rpm using a Thermomixer (Eppendorf) to enhance cell lysis. After extraction, genomic DNA was quantified using a Qubit® fluorometer (version 1.0, Invitrogen). Primer pairs 27f/1492r and DSR1F/DSR4R were used to amplify the 16 s rRNA and *dsrA* genes from *E. coli* and *D. vulgaris*, respectively, following PCR protocols described elsewhere [32], [35]. Standard PCR reactions were run with GoTaq® Green Master Mix (Promega), where 2 μL of appropriate genomic DNA served as the template. Amplicons were examined on a 1% agarose gel, and cleaned up using a QIAquick Gel Extraction Kit (Qiagen). Clean PCR products were quantified and appropriate ten-fold serial dilution series were constructed in TE buffer; the dilutions ranged from 4.75×10^8^ to 4.75×10^4^ targets μL^−1^ (*dsrA* standard series, r^2^ = 0.999) and 3.72×10^8^ to 3.72×10^4^ targets μL^−1^ (universal standard series, r^2^ = 0.994). All standards were stored at −20°C when not in use.

## Results

The present work was conducted to evaluate the effect of *Elodea nuttalli* rhizopshere on bacterial communities’ structure and MMHg production in Hg contaminated sediments. Three approaches were conducted to assess bacterial communities in sediments: T-RFLP profiling, sequencing of the 16S rRNA gene clone library and qPCR. T-RFLP profiling was conducted to analyze changes in the bacterial communities in rhizophere sediments of *E. nuttalli* compared to bulk sediments. Twenty-nine T-RFs were confidently identified in all replicates (n = 3). T-RFLP profiles in sediments were influenced by the presence of roots*:* six T-RFs (70 bp, 86 bp, 152 bp, 163 bp, 250 bp, 287 bp) were significantly decreased and four T-RFs (227 bp, 230 bp, 235 bp, 297 bp) were significantly increased in rhizospheric sediments compared to bulk sediments. PCA of T-RFLP profiles further confirmed differences between bulk and rhizospheric bacterial communities (Figure 1). Sequencing of 46 clones from the 16S rRNA gene library resulted in 31 OTUs in rhizospheric sediments whereas two clones could not be phylogenetically affiliated to the class level. *Deltaproteobacteria* dominated the rhizospheric bacterial community with up to 30% of the OTUs (Figure 2). Amongst the identified OTUs we found two *Desulfuromonale* -including one *Geobacteraceae*- and two *Desulfobacteraceae* as well as close relatives to iron-reducing bacteria (IRB; *Geobacteraceae, Rhodocyclaceae*), root associated nitrogen fixing bacteria (*Oxalobacteraceae*), denitrifying bacteria (*Anaerolineaceae, Steroidobactereae, Methylophilaceae*) and syntroph of SRB (*Syntrophaceae*; Figure 3 and 4).

Cloned sequences of P7 (HE648181) and P26 (HE648198) exhibited an experimental T-RF matching one of the T-RF (227 bp) induced in rhizospheric sediments compared to bulk sediments (Figure 5). However, the sequence analysis clearly showed that the two OTUs were two different phylotypes (Figure 3 and 4). P26 was phylogenetically affiliated to *Deltaproteobacteria*, most similar with a *Geobacter* sp. clone (GQ366586; 99% identity) and with a *Desulfuromonadales* bacterium clone (AM935526; 94% identity). P7 was affiliated to *Nitrospira* class related to a *Magnetobacterium* clone (EF613368; 96% identity) obtained from sulfate-reducing conditions and to a *Thermodesulfovibrio* clone (NR_041318; 85% identity). Because SRB were previously proposed to be the main actors of Hg methylation in this sediment, here we intended to assess if *E. nuttalli* roots could favour SRB in the sediments. We therefore quantified *drsA* and bacterial 16S rRNA genes using qPCR. However, data did not show any significant change in the proportion of *drs*A gene copies compared to total bacterial gene copies in rhizosphere sediments compared to bulk sediments (n = 6; Table 1).

To assess if the increase in potentially methylating bacteria could be evidenced on Hg methylation at the sediment level, we also measured total Hg and MMHg concentrations in sediments and pore water of rhizospheric and bulk sediments (n = 3; Table 2). Concentrations of _T_Hg and MMHg in pore water and sediments of bulk sediments were the same as at the beginning of the experiment (Table 2). In pore water of rhizosphere sediments _T_Hg concentrations decreased by 2-fold whereas MMHg concentrations increased slightly. As a result the percentage of MMHg of the _T_Hg in pore water increased 2.4-fold from 0.9% to 2.2%. In sediments, differences between bulk and rhizospheric sediments were not significant. Nevertheless a small decrease in _T_Hg was observed, with a small increase in MMHg from 0.22 to 0.25% of _T_Hg. We further wanted to assess if accumulation in plants could explain the decrease in dissolved _T_Hg. An increase of _T_Hg from the background concentration of 0.05±0.01 μg g^−1^ to 0.21±0.04 μg g^−1^ was observed in roots exposed to sediments. Concentrations of Hg in shoots and roots grown in the water column were 0.068±0.008 μg Hg g^−1^ and 0.054±0.010 μg Hg g^−1^ respectively. In all plant’s organs Hg was mainly found in the form of MMHg (100±5% of _T_Hg).

## Discussion

The aim of the present work was to assess the effect of a rooted macrophyte on Hg contaminated sediments and its bacterial community. *E. nuttallii* is an invasive plant originating from North America that is spreading through temperate climates all over the world. It is commonly found in heavy metal contaminated environments. Hg contaminated sediments were collected in a reservoir where high MMHg concentrations and extreme biomagnification in the food chain has been observed [19], [36]–[38]. Fish from Babeni reservoir are among the most contaminated fish found over the world with concentrations well above the threshold proposed by WHO [19], [36]–[38]. Moreover, concentration of _T_Hg found in the sediment is also well above the probable effect concentration (PEC: 1.06 μg g^−1^) previously proposed for sediments [39]. The toxicity of these sediments was confirmed by e.g. the absence of chironomids in the field [37], [38]. Field data suggested that SRB are the main methylators in sediments of this reservoir [19], [36]–[38]. Interestingly, the MMHg percentage of the _T_Hg significantly increased along the part of the reservoir in which macrophytes -including *Elodea nuttalli*- were found (upstream to middle part) [37], [38]. We consequently wondered if the presence of *E. nuttallii* could favor Hg methylation. Since methylation of Hg is thought to occur primarily through bacterial activity in sediments the present work was designed to assess the effect of *E. nuttallii* roots on bacterial communities and formation of MMHg in sediments.

Our data showed that roots of this plant significantly changed the bacterial community structure in this Hg contaminated sediments and increased the percentage of MMHg in pore water. An increase of MMHg concentrations has been observed in sediments colonized by macrophytes in salt marshes and wetlands where actively growing plants promoted Hg methylation in the sediments [15], [16]. Obviously, we cannot directly compare these works concerning salt marshes with our study. However, our data suggest that in a freshwater environment roots of *E. nuttallii* also increased MMHg production in rhizosphere, therefore it cannot be discarded that a longer exposure time in our study would have resulted in an increase of MMHg in whole sediments.

The detailed mechanistic understanding of Hg speciation is clearly outside the scope of the present work however, based on our data, it can be suggested that this species of plant affected the fate of Hg in sediments by i) reducing dissolved _T_Hg, ii) accumulating MMHg and iii) by creating an environment affecting microbiological activity and consequently Hg methylation. Indeed, although half the amount of _T_Hg was found dissolved in pore water of rhizospheric sediments, the concentration of MMHg slightly increased resulting in a significantly higher percentage of MMHg of the _T_Hg. Here, MMHg was internalized in roots while some inorganic Hg was likely either adsorbed to the root's surface or/and precipitated in sediments. Indeed, other authors have observed a precipitation of Hg in the rhizosphere of the marine macrophyte *Spartina* sp. [40]. Roots' exudates as well as microbes found on roots may exert a similar impact [40]. Such phenomenon cannot be excluded in our study and would obviously participate in a decrease of dissolved _T_Hg. Previously other authors also found a reduction of the pool of dissolved Hg in rhizospheric sediments, but they reported a demethylation effect [14]. Our data, on the opposite, suggested that MMHg concentration in pore water would likely increase if it was not accumulated by the roots and that *E. nuttallii* rather created a microenvironment favorable to Hg methylation. Notably, roots promoted the presence of two OTUs that could most probably be related to SRB and then Hg methylating bacteria, although further studies are needed to test the ability of these strain to methylate Hg. However, previous analysis carried out in the field also showed a predominance of SRB in Babeni reservoir sediments [37], [38].

Numerous studies have shown that bacteria play a role in mercury methylation, especially SRB [4], [6], [7]. Other studies, notably in the tropical Amazon environment have shown that MMHg production is very high in roots of floating macrophytes and could be linked to SRB activity [17], [18]. Increased sulfate-reducing activity has also been observed in the rhizosphere of marine and freshwater macrophytes [41], [42]. Other authors detected the presence of different SRB subgroups in macrophyte roots and observed differences in the composition and frequency of these groups between C_3_ and C_4_ plants [18]. Eventually an increase and a predominance of SRB in rhizosphere compared to bulk sediments was also observed [42]. Taken together, these publications suggest that SRB are often found in association with roots of marine and freshwater macrophytes. Favoring of SRB subgroup or species seem dependent on the plant species and its specific root exudates as well as sediment geochemistry. However, not all SRB are capable of mercury methylation [43]–[45] and other microorganisms, including IRB and methanogens may also be important [5], [46], [47].

Nowadays, the relative importance of each microbial group in the overall MMHg methylation/demethylation process is poorly understood in natural sediments [48]. Amongst SRB, *Desulfobacteraceae* were found to be very important mercury methylators in cultures [43], [45] and in periphyton [18], [49]. Amongst IRB, *Geobacter* sp. have also been shown to be able to methylate mercury [5], [46]. In the rhizosphere of *E. nuttallii*, several clones identified in our study are related to these different methylating bacteria and could therefore explain the effect of roots that increased MMHg, although the overall proportion of SRB was not different in bulk and rhizospheric sediments. More studies would be needed to link SRB to increased MMHg production in this environment [45]. However, the results of our study confirm that there is also a specific relationship between Hg methylation and the below-ground tissue of macrophytes in freshwater temperate environments as previously shown in marine, brackish or tropical environments.

A high occurrence of methylating bacteria species in macrophytes roots is relevant for many reasons. In the environment, a specific Hg contaminated site may be more conducive to one consortia of bacteria than others depending upon the macrophyte contributions, sediment geochemistry, and microbial interactions. Presence of *E. nuttallii* could result in higher occurrence of methylating bacteria and hence MMHg production and dispersion in the environment. In general, macrophytes play a role in the ecosystem notably as food source and shelter and consequently are densely populated by a varied fauna (e.g. invertebrates and fish). A high MMHg production in this environment may constitute a major pathway of MMHg uptake into aquatic food webs. *E. nuttallii* has been shown to bioaccumulate significantly MMHg from sediments and water while being at the base of the food chain [22], [37]. Last but not least *E. nuttalli* is constantly spreading and might result in the spreading of such favorable MMHg environments. In conclusion the presence of this plant in environments polluted by Hg should not be overlooked.
